# Instability of personal human metabotype is linked to all-cause mortality

**DOI:** 10.1038/s41598-018-27958-1

**Published:** 2018-06-28

**Authors:** M. E. Lacruz, A. Kluttig, D. Tiller, D. Medenwald, I. Giegling, D. Rujescu, C. Prehn, J. Adamski, K. H. Greiser, G. Kastenmüller

**Affiliations:** 1 Institute of Medical Epidemiology, Biostatistics and Informatics, Martin-Luther University Halle-Wittenberg, 06112 Halle, Germany; 2 Clinic of Psychiatry, Psychotherapy, and Psychosomatic, Martin-Luther University Halle-Wittenberg, 06112 Halle, Germany; 3 Institute of Experimental Genetics, Genome Analysis Center, Helmholtz Zentrum München, 85764 Neuherberg, Germany; 4 Lehrstuhl für Experimentelle Genetik, Technische Universität München, 85354 Freising-Weihenstephan, Germany; 5 German Center for Diabetes Research (DZD), 85764 Neuherberg, Germany; 6 German Cancer Research Centre, Division of Cancer Epidemiology, 69120 Heidelberg, Germany; 7 Institute of Bioinformatics and Systems Biology, Helmholtz Zentrum München, 85764 Neuherberg, Germany; 8 Department of Twin Research & Genetic Epidemiology, King’s College London, SE1 7EH London, UK

## Abstract

Disruption of metabolic homeostasis is an important factor in many diseases. Various metabolites have been linked to higher risk of morbidity and all-cause mortality using metabolomics in large population-based cohorts. In these studies, baseline metabolite levels were compared across subjects to identify associations with health outcomes, implying the existence of ‘healthy’ concentration ranges that are equally applicable to all individuals. Here, we focused on intra-individual changes in metabolite levels over time and their link to mortality, potentially allowing more personalized risk assessment. We analysed targeted metabolomics data for 134 blood metabolites from 1409 participants in the population-based CARLA cohort at baseline and after four years. Metabotypes of the majority of participants (59%) were extremely stable over time indicated by high correlation between the subjects’ metabolite profiles at the two time points. Metabotype instability and, in particular, decrease of valine were associated with higher risk of all-cause mortality in 7.9 years of follow-up (hazard ratio (HR) = 1.5(95%CI = 1.0–2.3) and 0.2(95%CI = 0.1–0.3)) after multifactorial adjustment. Excluding deaths that occurred in the first year after metabolite profiling showed similar results (HR = 1.8(95%CI = 1.1–2.8)). Lower metabotype stability was also associated with incident cardiovascular disease (OR = 1.2(95%CI = 1.0–1.3)). Therefore, changes in the personal metabotype might be a valuable indicator of pre-clinical disease.

## Introduction

Diseases linked to metabolic imbalance such as cardiovascular diseases (CVD) and diabetes are among the 10 leading causes of death in developed countries^1^. Metabolomic analyses, allowing the simultaneous quantification of more than 100 small-molecule metabolites in blood, provide a snapshot of the metabolic state of an organism. This capacity renders metabolomics particularly useful for studying the role of metabolic alterations in prevalent and incident disease, disease progression and mortality. For example, previous studies have found acylcarnitines, dicarboxylacylcarnitines, and various amino acids and lipid classes to associate with CVD morbidity^2^. Using different metabolomics platforms, several studies have identified metabolites that predict the occurrence of CVD^3–5^. Furthermore, applying a targeted metabolomics approach measuring 106 metabolic traits, Fischer *et al*.^6^ reported that four molecules, including citrate and various lipids were associated with all-cause and CVD mortality in a large European population-based cohort. Using non-targeted metabolomics technology in a cohort of African Americans, Yu *et al*.^7^ recently, identified nine metabolites from diverse metabolic pathways, such as steroids, bile acids, amino acids, dipeptides, and xenobiotics, that correlated with all-cause mortality.

In these studies, metabolite levels measured in samples from a single time point were used to test their association with prevalent and incident diseases or mortality, i.e., levels were compared across subjects to identify metabolites that indicate higher risk of disease or mortality if their levels are not within the ‘normal’ range (as defined by healthy individuals). In general, large studies analysing the change (i.e., increase or decrease) of metabolite levels over time within the same individuals are still sparse due to the lack of longitudinal metabolomics measurements^8^. However, studies investigating longitudinally collected multi-omics data for a smaller number of individuals have demonstrated the value of focusing on intra-individual changes of omics parameters over time, including metabolites, for personalized risk prediction^9,10^. For example, based on clinical tests, metabolome, proteome and microbiome data of 108 subjects assessed at three time points over 9 months, Price *et al*.^10^ generated a network showing the correlation of the changes between the analytes from one time point to the other. Interestingly, in this network, the metabolite gamma-glutamyltyrosine, a dipeptide, was directly connected with a variety of clinical parameters for cardiometabolic disease.

One underlying assumption when analysing changes of metabolite levels over time is that these levels are in principle stable, i.e., that they and their changes do not largely depend on short-term exposures. While levels of many metabolites such as those involved in energy metabolism or xenobiotics are highly dynamic and strongly influenced by, for example, fasting state^11^, numerous studies have shown that, overall, human metabolomes are stable and highly individual when compared over days and months^12–16^. Even when blood samples were drawn at several time points during metabolically demanding challenges such as physical exercise or a lipid-reach meal, the measured metabolomes (represented by the first three principal components of measured metabolite levels) clustered per subject^16^. Moreover, based on 212 metabolites in 818 subjects measured at two time points 7 years apart, we previously investigated the stability of metabolomes over time using correlation ranks of an individual’s metabolomes at baseline and follow-up as a measure of metabotype conservation^17^. Although the measured metabolomes included a variety of xenobiotics that are highly dynamic and highly influenced by specific short-term exposures such as food, we found that the personal metabolomes of the majority of participants (95%) in the population-based study were conserved over the 7-year period.

The primary goal of our present study was to investigate whether changes in the levels of metabolites over several years and the overall stability of the personal metabotype in this period are linked to subsequent cardiovascular events and all-cause mortality. To this end, we performed quantitative profiling of 163 metabolites, including acylcarnitines, amino acids, phospholipids and hexose, in blood samples from 1409 participants enrolled in the CARLA study at two time points separated by 4 years. Data on cardiovascular events and all-cause mortality were available for a mean follow-up time of 7.9 years from baseline.

## Results

This study is based on longitudinal serum samples from the German population-based CARLA cohort. Out of 1779 individuals examined at baseline, for 1409 participants, blood samples also were available for the follow-up visit after 4 years (Supplementary Figure 1). A total of 103 deaths occurred in this subsample within a mean follow-up time of 7.9 years (SD = 1.9) from baseline. Baseline characteristics of the study sample (n = 1409) by morbidity and mortality status are shown in Table 1. Being men, older and with prevalent diabetes were individually associated with mortality and cardiovascular events. Metabolomics measurements were performed in serum samples collected at baseline and at 4-years follow-up using the AbsoluteIDQ p150 kit for targeted mass spectrometry based quantification of 163 metabolites comprising mainly amino acids and lipids. After quality control based on reference samples (measured in >150 replicates), 29 metabolites were excluded (see Methods), leaving 134 metabolites for statistical analyses (Supplementary Table 1).

**Table 1:** Baseline characteristics of CARLA participants by mortality and morbidity status.

	Alive	Deceased participants	Healthy (no CVD) participants	Incident CVD
Total	1306	103	1167	115
Age: years, mean (SD)	62.6 (9.5)	71.8 (8.9)	62.2 (9.4)	66.4 (10.4)
Sex: female, N (%)	599 (45.9)	28 (27.2)	563 (48.2)	33 (28.7)
Diabetes, N (%)	152 (11.6)	24 (23.3)	130 (11.1)	20 (17.4)
Smoker, N (%)	243 (18.6)	18 (17.5)	228 (19.5)	24 (20.9)
BMI, mean (SD)	28.0 (4.5)	28.7 (5.1)	28.0 (4.6)	28.2 (3.8)
HDL cholesterol mmol/l, mean (SD)	1.4 (0.4)	1.3 (0.4)	1.4 (0.4)	1.3 (0.4)
Glucose mmol/l, mean (SD)	5.9 (1.7)	6.0 (1.6)	5.9 (1.7)	6.0 (1.4)
Triglyceride mmol/l, mean (SD)	1.9 (1.6)	2.0 (1.4)	1.8 (1.2)	2.6 (3.9)
Systolic BP mmHg, mean (SD)	143 (21)	145 (21)	143 (21)	147 (20)
Diastolic BP mmHg, mean (SD)	85 (11)	83 (12)	86 (11)	86 (12)
Follow-up years, mean (SD)	8.0 (1.9)	6.8 (1.6)	7.9 (1.9)	8.1 (1.6)

### Longitudinal changes in metabolite levels over 4 years and their association with mortality

**Figure 1:**
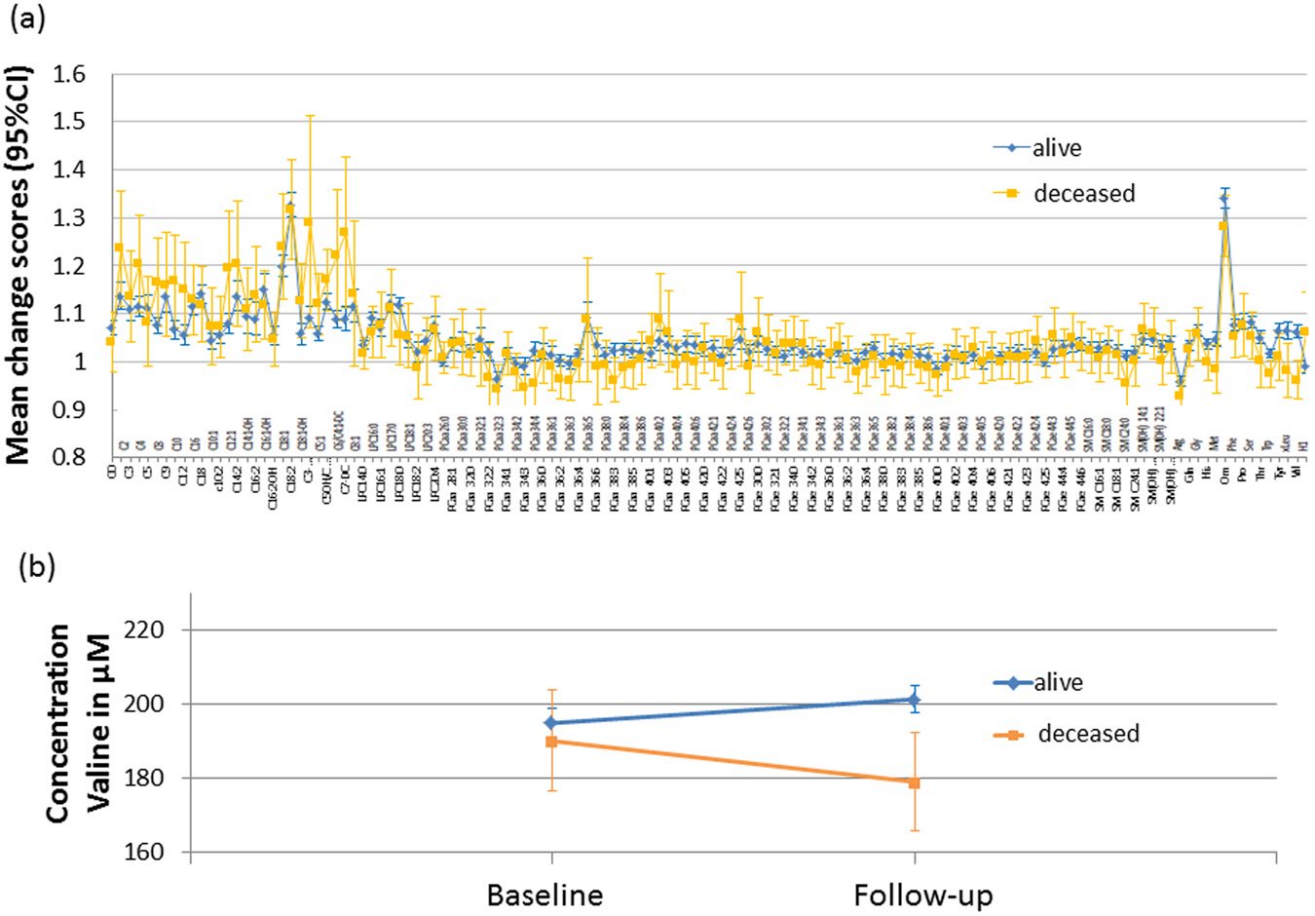
(**a**) Mean change (follow-up/baseline) and 95% CI in the individual metabolite concentrations stratified for vital status. (**b**) Mean concentrations of valine (μM) and 95% CI at baseline and follow-up stratified by vital status.

The intra-individual, longitudinal changes of metabolite levels over 4 years were assessed as the ratios between the follow-up and baseline concentrations of each participant. The majority of the quantified metabolites (123 out 134) showed a mean increase (follow-up/baseline ratio >1) (Fig. 1a) over time.

We then tested whether the longitudinal changes in metabolite concentrations associate with all-cause mortality using Cox regression. After Bonferroni correction for testing 134 metabolites, only the association of mortality and changes in the concentrations of the branched-chain amino acid valine remained significant (HR = 0.2, 95%CI = 0.1–0.3). 57% of the participants who were still alive after the maximum follow-up time showed an increase in valine concentrations within the 4 year period, whereas a decrease in valine concentrations was seen for 57% of the participants who died (χ^2^ = 7.1, p = 0.008) (Fig. 1b).

### Stability of the personal metabotype over 4 years and its association with mortality

The greater variance of metabolite changes observed in the group of subjects who died (Fig. 1a) suggests that the stability of an individual’s metabolome over time might be linked to mortality irrespective of the increase or decrease of specific metabolites. To test this hypothesis, we first assessed and investigated the stability of the personal metabolomes measured in our study cohort. To this end, we compared the intra-individual correlations between the quantitative metabolite profiles (based on 134 metabolites) at baseline and the 4-years follow-up for each participant with correlations between all inter-individual combinations of baseline and follow-up profiles. While, on average, the metabotypes of different individuals did not correlate (median of the longitudinal inter-correlations was −0.005 [n = 1 983 872 (each of the 1409 participants with each of the other 1408 participants), range −0.78 to 0.95]), the metabotypes of the same individual at baseline and 4 years follow-up showed high correlations with a median of 0.66 (n = 1409, range −0.09 to 0.93), indicating the high conservation of personal metabotypes over 4 years.

**Figure 2:**
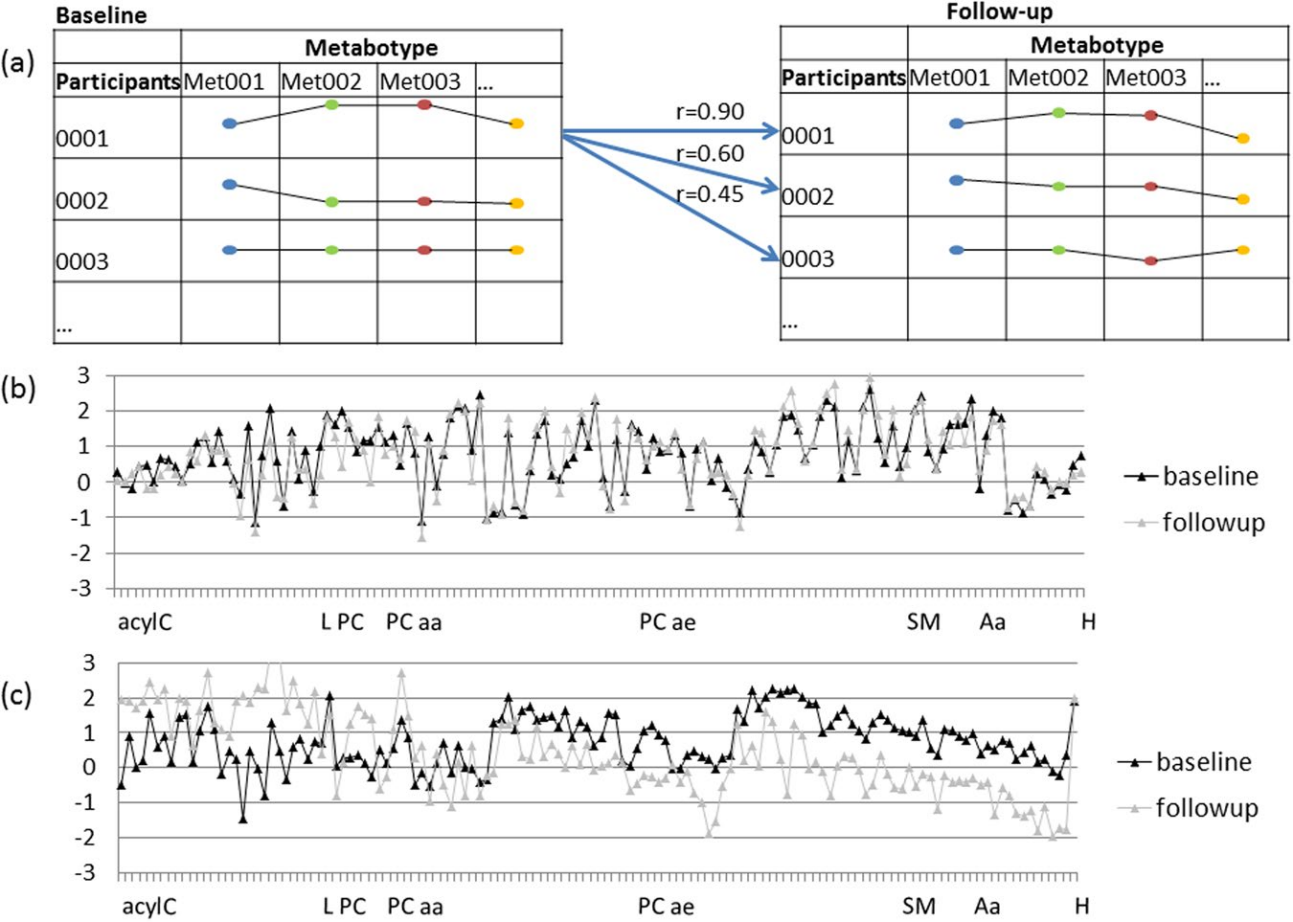
(**a**) Description of the calculation of the conservation index (long-term stability of individual profiles). The correlations of each baseline metabotype with all other metabotypes at follow-up, are converted to ranks to measure a metabotype’s similarity to itself when compared to its similarity to other metabotypes. The conservation index is calculated as 1 − ((rank(i) − 1)/(N − 1)), where N is the number of metabotypes and (i) is each participants. This index quantifies the comparison of intra-correlations to inter-correlations, yielding a value in the range [0,1]. Log- and standardised metabolite concentrations for baseline (black lines) and follow-up (grey dotted lines) of a participant with high conservation index = 1 (**b**) and another with low conservation index = 0.62 (**c**).

As a measure of metabotype persistence over time, we used the so-called metabotype conservation index, a rank-based measure introduced by Yousri *et al*.^11^ to quantify the stability of metabotypes that were assessed on a non-targeted metabolomics platform. The metabotype conservation index of an individual is defined as the relative rank of the longitudinal metabotype intra-correlation of this individual with respect to all longitudinal metabotype inter-correlations of this individual with all other individuals from the same study cohort (Fig. 2a). Accordingly, a conservation index of 1 indicates the high stability of an individual’s metabotype (Fig. 2b), while a conservation index around 0 indicates low stability (Fig. 2c). In the CARLA-cohort, a conservation index value of 1 was observed for 829 out of the 1409 participants, i.e. 59% of the study participants could be uniquely identified in this population after 4 years based only on metabolic profile information. Furthermore, 97% of the conservation index were above 0.9 (n = 1360) and 99% above 0.7 (n = 1392) i.e., the intra-correlation over time was ranked among the 30% highest correlations with all other individuals (Supplementary Figure 2), indicating the generally high stability of personal metabolomes over years in our study population. We thereby replicated previous findings of long-term metabotype stability^17^ that were based on fasting samples and a broad non-targeted metabolomics profiling in an independent non-fasting cohort using a lipid-focused targeted metabolomics panel.

**Figure 3:**
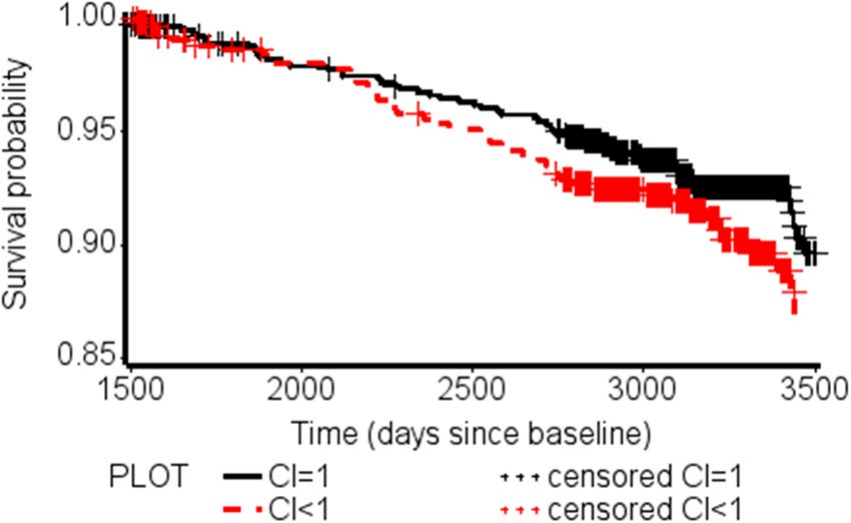
Kaplan-Meier curves stratified by conservation index (“conservation index” = 1 in black and “conservation index” < 1 in red).

Using the conservation index as a measure for metabotype stability, we finally compared the mortality risk of participants with high metabotype stability (conservation index = 1) with the risk of those participants with lower stability (conservation index <1). Individuals with lower metabotype stability had a 1.5 fold increased risk for all-cause mortality (HR = 1.52, 95%CI = 1.01–2.26) (Table 2 and Fig. 3). In order to reduce confounding due to existing illnesses or processes at the time of the 4-years follow-up blood draw, we ran a sensitivity analysis excluding those participants who died during the first year of follow-up. These exclusions reduce the possible effect of reverse causality. In this analysis, with 84 participants who died after that period, we obtained very similar effects as in the original analysis without exclusions: HR = 1.76, 95%CI = 1.12–2.77.

**Table 2:** Mortality rate per 1000 person-years and hazard ratios (HR) with 95% CI for the association of conservation index with all-cause mortality among CARLA participants, 2002–2013.

Conservation index (Deaths/Person-Years)	Mortality rate (per 1000 Person-Years) with 95%CI	HR	95% CI
**All-cause mortality (n** = **103/1409)**			
1 (55/6577)	8.4 (6.4–10.9)	Ref	—
<1 (48/4576)	10.5 (7.9–13.9)	1.52	1.01–2.26
**Sensitivity analysis: all-cause mortality at least one year after follow-up (n = 84/1168)**			
1 (44/5983)	7.4 (5.5–9.9)	Ref	—
<1 (40/4175)	9.6 (7.0–13.1)	1.76	1.12–2.77

For the prediction of all-cause mortality, a model containing known life-style CVD risk factors (Body Mass Index (BMI), alcohol consumption, smoking, diet and physical activity) and the conservation index only showed a modest improvement (Receiver Operating Curve area under the curve (ROC-AUC 0.70) compared with the model containing known CVD risk factors alone (AUC ROC 0.69). Similarly for the prediction of CVD mortality, the model containing CVD risk factors and the conservation index showed a modest improvement (ROC-AUC 0.73) compared with the model containing known CVD risk factors alone (AUC ROC 0.72).

### Links between metabotype stability and CVD

Out of 1282 participants free of CVD at baseline 115 participants developed CVD during the follow-up time of 7.9 years. The risk of self-reported incident CVD did not significantly differ between the group of participants with high metabotype stability (conservation index = 1) and the group with lower stability (conservation index <1) (OR = 1.02, 95%CI 0.99–1.05) (Table 3). However, a further differentiation of those participants with a low conservation index in two groups, namely in participants with a medium conservation index (between 0.7 and 1) and participants with a very low conservation index (equal or below 0.7), showed an association between conservation index and incident CVD (OR = 1.15, 95%CI 1.01–1.30).

**Table 3:** Odds ratios (OR) for the association of conservation index with incident CVD among CARLA participants, 2002–2013.

Conservation index	healthy/ill	OR	95% CI
1	696/63	Ref.	—
<1	471/52	1.02	0.99–1.05
1	696/63	Ref.	—
1< and >0.7	459/48	1.01	0.98–1.05
**≥0.7**	**12/4**	**1.15**	**1.01–1.30**

**Figure 4:**
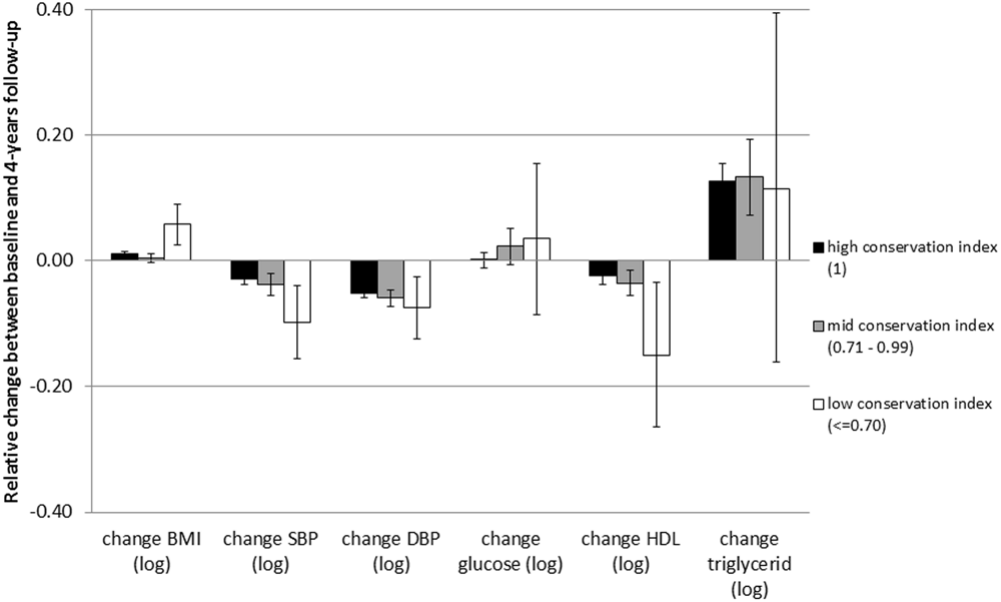
Relative mean changes and 95% CI in cardiovascular risk factors (logarithmized scores) for participants with a conservation index of 1 (black), between 0.7 and 1 (grey), and equal or below 0.7 (white).

We further investigated the association of metabotype conservation with several CVD risk factors, such as BMI, blood pressure and serum lipids. Those participants with a conservation index equal or below 0.7 had a greater relative increase in BMI (Kruskal-Wallis Test χ^2^ = 11.4, p < 0.005) and almost significant decreases in systolic blood pressure (SBP) (Kruskal-Wallis Test χ^2^ = 5.8, p = 0.06) and in HDL-cholesterol concentrations (Kruskal-Wallis Test χ^2^ = 5.8, p = 0.05) from baseline to 4-years follow-up than those with higher conservation index. No differences could be seen for longitudinal changes in the CVD risk factors diastolic blood pressure, glucose and triglycerides (Fig. 4).

Closer inspection of the 17 participants (1%) with a very low conservation index (≤0.7) based on additionally available clinical and lifestyle parameter showed that 13 out of the 17 subjects started a CVD therapy between baseline and the 4-years follow-up or changed their CVD therapy during this period (Table 4). For two of the remaining participants medications indicated an acute infection at one of the two time points. For two participants, we did not find any immediate explanation for the large change in their metabotype between baseline and 4-years follow-up and the accordingly low intra-individual correlation between the metabolite profiles at the two time points (Table 4, Subject 1: r = −0.1; Subject 12: r = 0.2).

**Table 4:** Characteristics of CARLA participants with low conservation index.

**Subject**	**CI**	**Sex**	**Age**	**Alcohol** ^*^	BMI*	**Smoker**	SBP**	**DBP** ^***^	**CVD**	**T2D**	**CVD med**	**med baseline**	**med followup**	**Acute infection**	Glucose*	HDL-chol*	Tg*
**1**	0.7	♂	50	↑		↑						none	none	Yes			↓
**2**	0.5	♂	55				↓	↓			↑	none	b, l			↓	
**3**	0.5	♂	64	↑			↓	↓		↑		b, d, l, v	a, b, d, l, v				
**4**	0.7	♂	63	↓							↑	none	a, b		↓		↓
**5**	0.7	♂	69				↓	↓		↑		b, l	a, b, l		↓		
**6**	0.4	♂	76	↓			↓	↓				c	v				
**7**	0.7	♂	75				↓	↓				b, d, l, v	a, b, c, d, v			↓	↑
**8**	0.6	♀	49									b, c, g	b, c, l			↓	↓
**9**	0.7	♀	53	↑							↑	none	b				↑
**10**	0.4	♀	51	↓			↓	↓			↑	none	a			↑	↑
**11**	0.1	♀	57					↓				none	none				↑
**12**	0.7	♀	57	↓			↑					none	none	Yes		↓	
**13**	0.6	♀	58				↓	↓				a, b	a, b, c				↑
**14**	0.7	♀	65	↓								none	none				
**15**	0.3	♀	65	↓			↓	↓			↑	none	b, v				
**16**	0.7	♀	64	↓								l	a, d, l		↑		↓
**17**	0.6	♀	80				↓	↑	↑			a, b, c, v	a, b, c, d, l, v			↓	↑

*Changes <20% empty field, increases >20% arrow upwards and decreases >20% arrow downwards.

**Changes <10 mmHg empty field, increases >10 mmHg arrow upwards and decreases >10 mmHg arrow downwards.

***Changes <5 mmHg empty field, increases >5 mmHg arrow upwards and decreases >5 mmHg arrow downwards.

CI = conservation index; SBP = systolic blood pressure; DBP = diastolic blood pressure; CVD = cardiovascular disease; T2D = diabetes mellitus, med = medication; HDL-chol = HDL-cholesterol; Tg = triglycerides; a = angiotensin-converting-enzyme -inhibitors; b = beta-blocking agents; c = calcium channel blockers; d = diuretics; g = angiotensin receptor blockers; l = lipid modifying agents; v = vasodilator.

## Discussion

In the present study, we focused on the association of intra-individual, longitudinal changes in metabolism with health outcomes, namely incident CVD and all-cause mortality rather than investigating their associations with inter-individual metabolic differences. To this end, we inspected longitudinal metabolomics data in blood serum from 1409 subjects of the prospective, population-based CARLA cohort at baseline and a 4-years follow-up examination in relation to their morbidity and mortality outcomes over a follow-up time of 7.9 years on average.

### Decrease of valine over four years associates with all-cause mortality

Analysing the association of all-cause mortality with intra-individual changes between baseline concentrations and concentrations at the 4-years follow-up for 134 measured metabolites (mainly amino acids and lipids), we found that a decrease of valine levels over the four year period was linked to higher risk of mortality in the following 3.9 years. In various previous cross-sectional studies, lower levels of branched-chain amino acids (BCAA) and valine have been associated with prevalent and incident Alzheimer’s disease (AD)^18,19^ as well as higher mortality risk^20^. Our results demonstrate that not only lower blood levels of valine per se but also the decrease of valine concentrations over time, irrespective of the individual baseline levels, indicates higher mortality risk. Considering the importance of BCAA for muscle tissue, the observed decrease in valine concentrations might be a sign of loss in lean body mass (sarcopenia) causing frailty. In case of a causal relationship - which certainly cannot be concluded from any association analysis as the one presented here – supplementation of valine might attenuate adverse effects of low BCAA levels related to muscles. Interestingly, beneficial effects of BCAA supplementation have been shown in animal studies. For instance, D’Antona and colleagues examined the effects of a diet supplemented with BCAA in mice and demonstrated that the function of cardiac and skeletal muscles improved and the average lifespan increased in comparison with mice without supplementation^21,22^. However, in humans the benefit of BCAA supplementation is less evident^23,24^. A possible reason might be that not only low levels of BCAAs associate with adverse health outcomes: In contrast to AD and mortality, higher levels of valine and further BCAA have been linked to prevalent and incident type 2 diabetes (T2D)^25,26^ and incident CVD in women^27^. Thus, balancing the potential positive effects of BCAA supplementation with potential adverse effects^28^ might be crucial.

### Personal metabotypes are stable over years in observational studies

While metabolite levels can change significantly within a subject over minutes, hours and days on response to external stimuli such as food^16^, it has been shown that individuals usually stay within their own ‘metabolic space’ (metabotype) when taking the perspective of the entire metabolome (typically sketched by all metabolites measured in a metabolomics analysis). Nonetheless, the personal metabotype can be altered over a longer period as a consequence of large changes in lifestyle or targeted lifestyle interventions^29^. However, as shown by our previous study such alterations in the personal metabotype were rare in the case of an observational study without interventions: in the German population-based KORA cohort, only 5% of 818 participants showed low intra-individual correlation of their fasting blood metabolite profiles at baseline and a 7-years follow-up examination, indicating an overall high stability of personal metabotypes^17^.

In the present study, we have replicated these previous findings about long-term stability of metabotypes in the independent CARLA cohort (n = 1409) for metabolite profiles at baseline and a 4-years follow-up visit using the same correlation-based measure for metabotype conservation, the conservation index, as Yousri and colleagues^17^. Compared to the results in the KORA cohort, we found an even lower percentage of participants with low correlation between baseline and follow-up profiles of 1% though CARLA blood samples were drawn in a non-fasting state. The shorter time of four versus seven years between baseline and follow-up examination could be one possible reason for this observation. Moreover, in CARLA, we used a targeted metabolomics platform with the majority of measured metabolites being phospholipids, which are less influenced by fasting status compared to the metabolites assessed by the broad metabolic profiling applied in KORA, which also includes xenobiotics. Detailed inspection of medication data and lifestyle parameters available for the 17 individuals with very low metabotype stability (conservation index ≤0.7) over four years in CARLA, revealed a start or change of drug treatment possibly explaining the large changes in the personal metabotype of 13 participants. Interestingly, two participants out of the remaining four subjects took medication indicating acute infections at one of the two time points. This observation of large changes in the metabolome on response to an infection is in line with previous findings in Chen *et al*.^9^ where the authors recorded multi-omics profiles for a subject over 14 months including a phase of infection.

### Instability of personal metabotype is linked to all-cause mortality and incident CVD

Considering the high conservation of a person’s entire metabotype over years, we here were not only interested in how changes in individual metabolites affect health outcomes, but also in the role of general metabotype stability irrespective of particular metabolites. In the middle-aged (45–65 years) and elderly (65 and older) CARLA population cohort, we found that less stable serum metabotype (conservation index <1) was associated with a 50% increase (HR = 1.5) in all-cause mortality risk over 8 years of follow-up. This association was independent of several health parameters (cardiovascular risk factors and chronic illness). Moreover, greater changes in risk factors (BMI, SBP (systolic blood pressure) and HDL) were associated with lower conservation index, thus, supporting our hypothesis that metabotype instability is associated with hard-health endpoints (laboratory findings and mortality). Furthermore, severe metabotype changes (conservation index ≤0.7) were associated with self-reported incident CVD. As we could see, the most relevant changes in those individuals were associated with cholesterol levels. Indicating that those participants with lower conservation index had probably changes in the lipid metabolites either due to a CVD (including hypertension) or the therapy applied to it.

Associations between metabotype instability and mortality remained significant when we excluded deaths occurring in the first year after the 4-years follow-up examination (time point of the second metabolomics measurement) to avoid influences from expectable metabolic shifts in case of prevalent severe illness. Also adjustment for renal function using creatinine concentrations did not change the association in the original and sensitivity analysis neither for the complete data set nor for men or women.

Our results therefore suggest that monitoring changes in an individual’s metabotype can trace decline in health status without focusing on biomarkers for any specific disease.

### Strengths and limitations

The strengths of this study include the use of a large representative population cohort with multiple health parameters. Further strengths are that samples from both time points were measured at the same time aleatory mixed and that targeted metabolomics provided absolute metabolite concentrations. These conditions make the longitudinal metabotypes much better comparable than in previous studies.

The main limitation in this study is inherent to all observational studies, which do not allow us to draw causal inferences. Our results, therefore, may be due to reverse causality or due to unmeasured confounders. Also, some variation may have resulted from laboratory errors associated with sample storage and variation in the time of day at which the samples were collected at each examination. A further limitation is the non-fasting state of the study population. It is well-known that fasting status influences the concentrations of several of the metabolites measured with the Absolute*IDQ* p150 Kit, in particular the amino acids and carnitines^16^. However, it seems that the conservation index is not or only minimally influenced by fasting status, as the proportion of participants with high conservation index in our cohort is even higher than in the previous study based on blood samples collected after overnight fasting^17^. Moreover, o*nly Caucasian* patients were enrolled in CARLA, hampering extrapolation of our results to other ethnic groups. In addition, the association between conservation index and health outcomes found in our study have not been confirmed in a validation cohort yet.

## Conclusions

We studied the long-term conservation of metabotype profiles over 4 years and its association with health outcomes. The findings of this study show that an unstable metabotype is associated with higher all-cause mortality and CVD morbidity in humans and indicate that this effect is also associated with changes in classical CVD risk factors. This makes metabotype monitoring over time relevant for early diagnosis and prevention of serious health problems without the need to focus on specific disease biomarkers.

## Methods

### Study population

The CARLA-Study is a population-based cohort study in the city of Halle/Saale in eastern Germany. Study design and methods were described in detail elsewhere^30^. In brief, at baseline 1779 subjects (46% women) aged 45 to 83 years were examined between July 2002 and January 2006 (response rate 64%). After a mean of 4 years (SD = 0.3) 1436 subjects (45% women) took part in the follow-up examination (response rate 86%). The present study included a total of 1409 participants, who had metabolite measurements at both time points (see Supplementary Figure S1 for flow-chart of the study). No sex-distribution differences were seen among participants excluded and included, but those excluded from this analysis tended to be older. The CARLA study was carried out in accordance with the declaration of Helsinki. All participants gave their written informed consent. The study was approved by the local ethics commission of the Medical Faculty of the Martin-Luther University Halle-Wittenberg.

### Metabolite profiling

For this study, we measured the blood metabolomic profiles of the study participants. Non-fasting blood samples were taken after supine rest (30 minutes). After 10-min centrifugation (20 °C, 1500 RPM), the serum was collected, and after a clotting time of 30 minutes, deep frozen to −80 °C within one and half hours and stored until analysis of the metabolites.

Metabolomic profiles in the blood serum were analysed using the Absolute*IDQ* p150 kit (Biocrates Life Sciences AG, Innsbruck, Austria) and flow injection analysis-tandem mass spectrometry (FIA-MS/MS). Out of 10 µL serum, 163 metabolites were quantified, including amino acids (AA), acylcarnitines (acylC), hexoses, phospho- (PC) and sphingo-lipids (SM). For a full-list of all quality-controlled metabolites, see Supplementary Table S1). The method of Absolute*IDQ* p150 kit has been proven to be in conformance with the EMEA-Guideline “Guideline on bioanalytical method validation”^31^ which implies proof of reproducibility within a given error range. The assay procedures of kit as well as the metabolite nomenclature have been described in detail previously^32,33^.

Sample handling was performed by a Hamilton MicrolabSTAR robot (Hamilton Bonaduz AG, Bonaduz, Switzerland) and a Ultravap nitrogen evaporator (Porvair Sciences, Leatherhead, U.K.), beside standard laboratory equipment. Mass spectrometric analyses were done on an API 4000 triple quadrupole system (Sciex Deutschland GmbH, Darmstadt, Germany) equipped with a 1200 Series HPLC (Agilent Technologies Deutschland GmbH, Böblingen, Germany) and a HTC PAL auto sampler (CTC Analytics, Zwingen, Switzerland). Autosampler and mass spectrometer were controlled by the software Analyst 1.6.2 (Sciex Deutschland GmbH, Darmstadt, Germany). Data evaluation for quantification of metabolite concentrations and quality assessment were carried out using the Met*IDQ* software package (Biocrates Life Sciences AG, Innsbruck, Austria). Quantification of the metabolites was achieved by reference to the appropriate internal standards. Metabolite concentrations are reported as μmol/L. Five reference plasma samples were measured with each batch of samples and were used to determine the reproducibility of the assay.

### Quality control of the metabolomics dataset

Two metabolites (lysoPC a C6:0 and PC ae C38:1) were excluded as the number of values below the limit of detection (LOD) within lab analysis exceeded 5% (values = 0). The remaining missing values (1‰ of all values) were imputed using the multiple imputation SAS procedure with the MCMC (Markov chain Monte Carlo) method. Imputations were done with minimum and maximum values defined from the CARLA population and every imputation was plausibility checked. A coefficient of variation (CV) was calculated for each metabolite based on 173 aliquots of a reference plasma sample measured together with the CARLA samples (5 on each plate). 27 metabolites for which the CV exceeded 25% were excluded from further analysis (13 acylC, 9 PC and 5 SM). As the metabolic profile was assessed on thirty-five plates, a so called batch variable was included in the analyses as a random factor in order to avoid possible effects due to technical issues or different time of measurement. No outliers, defined as greater than mean ± 5 standard deviations of the particular metabolite over the whole population, were found^34^.

### Outcome

We obtained mortality data from the population registry and incident disease data from the CARLA follow-up examinations. The vital status of study participants was followed from baseline through 2013 (8-years of follow-up in mean) via the population registry. Incident CVD was defined based on self-reported physicians’ diagnosis within the follow-up period (4- and 8-years follow-ups). Cardiovascular disease included myocardial infarct (MI), coronary artery bypass graft (CABG), percutaneous transluminal coronary angioplasty (PTCA), stroke, and carotid surgery.

### Confounders

Baseline and follow-up assessments included a personal interview, medical examinations and questionnaires. Sociodemographic (age and sex), life-style factors (alcohol consumption and smoking habits) and health-related outcomes (self-reported diabetes and use of medication within the last week) were collected at the interview. CVD medication was automatically coded according to the Anatomical Therapeutic Chemical Classification System (ATC codes C01–C10). The medical examination included anthropometric, blood pressure measurements and blood collection for laboratory measurements. The determination of creatinine was undertaken by the Institute of Laboratory Medicine, Clinical Chemistry and Molecular Diagnostics at Leipzig University Clinics.

### Conservation index

To quantify the similarity of a person’s metabotype at baseline to his/her metabotype at follow-up, we calculated a (metabotype) conservation index for each person following Yousri *et al*.^17^. Thereby a metabotype is defined as the whole set of metabolite concentrations measured for a particular blood sample, i.e., in our case consisting of 134 metabolites (134-tupel). For each individual (‘*i’)* in the study, we first calculated the Pearson’s correlations between its metabotype at baseline and the metabotypes at follow-up of all N = 1409 individuals (‘*j’)* (*j* = 1, …, N) in the study. We then ranked the baseline(*i*)-follow-up(*j*) pairs according to their Pearson’s correlation in decreasing order with *rank(i)* denoting the rank of the baseline(*i*)-follow-up(*i*) pair in this list. The *conservation index* is defined as the relative rank of the longitudinal metabotype intra-correlation of each individual (=*rank(i)*) with respect to the inter-correlations between the individual’s baseline metabotype and the follow-up metabotypes of all other individuals from the same study cohort calculated as 1 − ((*rank*(*i*) − 1)/(*N* − 1)). The conservation index yields values in the range between 0 (low similarity, i.e. low conservation of metabotype over time) to 1 (high similarity, i.e., high conservation of metabotype over time). Figures 2b,c show examples of participants with high and low conservation index.

### Statistical analysis

All analyses were performed with SAS 9.4 (SAS Inc., Cary, NC, USA). Associations between all-cause-mortality and (i) changes in individual metabolites (assessed as ratios between their follow-up and baseline concentrations) or (ii) the conservation index were identified using Cox propotional hazards models (*Proc Phreg*), with adjustment for sex, age, BMI, systolic and diastolic blood pressure, CVD medication, HDL cholesterol levels and triglycerides and creatinine concentrations, diabetes, smoker status and alcohol consumption at baseline and 4-years follow-up. To account for multiple testing when analysing the metabolites separately, Bonferroni correction was applied considering p-values with p < 0.05/134 as significant. To account for sex differences, we included interaction terms between conservation index and sex in those models where the p-value of the interaction was below p < 0.20. There was no effect of the interaction; therefore we report the results of the model without interaction. The proportional hazards assumption was tested using Schoenfeld residuals and the residuals did not significantly deviate from zero slope. Because metabotypes that are measured only shortly before the event could be influenced by undiagnosed morbidity, we ran sensitivity analyses excluding deaths that occurred in the first year after the 4-years follow-up. This sensitivity analysis reduces the possible effect of reversed causality. To assess the value of the conservation index for the prediction of all-cause mortality, we computed the area under receiver operating curves (ROC-AUC) for a model with classical CVD risk factors (alcohol, smoking, BMI, sport and diet) and for a model that included classical risk factors and the conservation index (*Proc logistic*). To estimate the effect of metabotype conservation as quantified by the conservation index, on incident CVD, we used logistic regression models (*Proc Genmod*). Confounders taken into account for this association were: sex, age, BMI, smoker, alcohol consumption, CVD medication, diabetes and creatinine levels at baseline and 4-years follow-up. Differences in CVD risk-factor changes between three conservation index groups (conservation index = 1, 0.7 < conservation index < 1, conservation index ≤ 0.7) were analysed with the Kruskal-Wallis test (*Proc Npar1way*).

### Data availability

Data are subject to ethical and national data protection laws and are available only through an individual project agreement with CARLA. Requests should be sent to alexander.kluttig(at)medizin.uni-halle.de and are subject to approval by the CARLA Steering Committee (http://www.medizin.uni-halle.de/index.php?id=1109).

## Electronic supplementary material

Supplementary information
