# Supplementary material for: Instability of personal human metabotype is linked to all-cause mortality
Source: Sci Rep. 2018 Jun 28;8:9810. doi: 10.1038/s41598-018-27958-1 (PMC6023858; doi:10.1038/s41598-018-27958-1)
Supplement: Supplementary file 1 — Supplementary information [file 41598_2018_27958_MOESM1_ESM.docx]

**Instability of personal human metabotype is linked to all-cause mortality**

Lacruz ME, Kluttig A, Tiller D, Medenwald D, Giegling I, Rujescu D, Prehn C, Adamski J, Greiser KH, Kastenmüller G

Supplementary Figure 1. Flow chart of the CARLA study

Baseline examination

July 2002 – Jan 2006

N=1779

Died (n=105)

Unreachable (n=7)

Excluded (n=108): address unknown or severe illness

Declined participation (n=123)

4-years follow-up

Mar 2007 – Mar 2010

N=1436

Exclusion participants without
blood sample (n=27)

Current analysis

N=1409

Supplementary Figure 2. Metabotype conservation index: The conservation index of the metabotype of a study participant is defined as the relative rank of the longitudinal intra-correlation of the metabolic profile of that individual compared to the longitudinal inter-correlations with the profiles of all other study participants. In this sub-cohort of the CARLA study (n=1409), 99% of the participants had a metabotype conservation index above 0.70.

Supplementary table 1. List of metabolites (N=134) with raw values (before transformation) and correlation between baseline and follow-up values per metabolite (n=1409 participants).

| **Biochemical name** | **Short name** | **Metabolite class** | **Baseline** | | **Follow-up** | | **r (Pearson)** |
| --- | --- | --- | --- | --- | --- | --- | --- |
|  |  |  | **Mean** | **Std Dev** | **Mean** | **Std Dev** |  |
| Carnitine | C0 | Acylcarnitines | 45.981 | 12.328 | 47.890 | 12.829 | 0.61 |
| Acetylcarnitine | C2 | Acylcarnitines | 8.199 | 3.670 | 8.502 | 3.514 | 0.46 |
| Propionylcarnitine | C3 | Acylcarnitines | 0.457 | 0.200 | 0.481 | 0.223 | 0.51 |
| Butyrylcarnitine | C4 | Acylcarnitines | 0.247 | 0.113 | 0.268 | 0.150 | 0.70 |
| Valerylcarnitine | C5 | Acylcarnitines | 0.169 | 0.060 | 0.178 | 0.077 | 0.42 |
| Tiglylcarnitine | C5:1 | Acylcarnitines | 0.039 | 0.010 | 0.040 | 0.011 | 0.47 |
| Octanoylcarnitine | C8 | Acylcarnitines | 0.151 | 0.061 | 0.158 | 0.074 | 0.60 |
| Octenoylcarnitine | C8:1 | Acylcarnitines | 0.151 | 0.076 | 0.151 | 0.084 | 0.48 |
| Nonaylcarnitine | C9 | Acylcarnitines | 0.042 | 0.025 | 0.044 | 0.027 | 0.61 |
| Decanoylcarnitine | C10 | Acylcarnitines | 0.249 | 0.104 | 0.255 | 0.120 | 0.56 |
| Decenoylcarnitine | C10:1 | Acylcarnitines | 0.135 | 0.049 | 0.134 | 0.047 | 0.55 |
| Decadienylcarnitine | C10:2 | Acylcarnitines | 0.052 | 0.014 | 0.053 | 0.019 | 0.39 |
| Dodecanoylcarnitine | C12 | Acylcarnitines | 0.112 | 0.045 | 0.114 | 0.048 | 0.57 |
| Dodecenoylcarnitine | C12:1 | Acylcarnitines | 0.107 | 0.044 | 0.110 | 0.045 | 0.62 |
| Tetradecadienylcarnitine | C14:2 | Acylcarnitines | 0.022 | 0.011 | 0.022 | 0.010 | 0.46 |
| Hexadecanoylcarnitine | C16 | Acylcarnitines | 0.131 | 0.041 | 0.140 | 0.041 | 0.55 |
| Hexadecadienoylcarnitine | C16:2 | Acylcarnitines | 0.009 | 0.003 | 0.009 | 0.004 | 0.32 |
| Octadecanoylcarnitine | C18 | Acylcarnitines | 0.056 | 0.018 | 0.060 | 0.019 | 0.56 |
| Octadecenoylcarnitine | C18:1 | Acylcarnitines | 0.137 | 0.048 | 0.155 | 0.051 | 0.51 |
| Octadecadienylcarnitine | C18:2 | Acylcarnitines | 0.043 | 0.016 | 0.053 | 0.018 | 0.50 |
| Hydroxybutyrylcarnitine | C4.OH (and C3-DC) | hydroxy- and dicarboxy- acylcarnitines | 0.073 | 0.051 | 0.074 | 0.049 | 0.54 |
| Hydroxyvalerylcarnitine | C5.OH (and C3.DC.M) | hydroxy- and dicarboxy- acylcarnitines | 0.037 | 0.010 | 0.040 | 0.011 | 0.55 |
| Hexanoylcarnitine | C6 (and C4.1.DC) | hydroxy- and dicarboxy- acylcarnitines | 0.088 | 0.030 | 0.093 | 0.036 | 0.53 |
| Pimeloylcarnitine | C7-DC | hydroxy- and dicarboxy- acylcarnitines | 0.035 | 0.016 | 0.036 | 0.016 | 0.53 |
| Hydroxytetradecenoylcarnitine | C14:1-OH | hydroxy- and dicarboxy- acylcarnitines | 0.014 | 0.005 | 0.014 | 0.008 | 0.30 |
| Hydroxyhexadecenoylcarnitine | C16:1-OH | hydroxy- and dicarboxy- acylcarnitines | 0.011 | 0.004 | 0.012 | 0.005 | 0.33 |
| Hydroxyhexadecadienoylcarnitine | C16:2-OH | hydroxy- and dicarboxy- acylcarnitines | 0.012 | 0.003 | 0.013 | 0.004 | 0.37 |
| Hydroxyoctadecenoylcarnitine | C18:1-OH | hydroxy- and dicarboxy- acylcarnitines | 0.012 | 0.003 | 0.012 | 0.004 | 0.35 |
| Arginine | Arg | Amino acids | 122.287 | 22.789 | 115.144 | 22.550 | 0.52 |
| Glutamine | Gln | Amino acids | 587.673 | 108.511 | 598.347 | 108.831 | 0.57 |
| Glycine | Gly | Amino acids | 259.273 | 82.561 | 268.518 | 80.542 | 0.74 |
| Histidine | His | Amino acids | 82.465 | 22.201 | 83.865 | 25.353 | 0.77 |
| Methionine | Met | Amino acids | 31.913 | 10.089 | 32.072 | 7.890 | 0.37 |
| Ornithine | Orn | Amino acids | 69.145 | 19.210 | 88.825 | 23.463 | 0.47 |
| Phenylalanine | Phe | Amino acids | 55.161 | 11.234 | 58.107 | 11.830 | 0.47 |
| Proline | Pro | Amino acids | 235.499 | 78.320 | 246.019 | 80.940 | 0.63 |
| Serine | Ser | Amino acids | 110.999 | 26.838 | 117.290 | 27.427 | 0.64 |
| Threonine | Thr | Amino acids | 109.209 | 30.507 | 110.878 | 30.473 | 0.57 |
| Tryptophan | Trp | Amino acids | 87.181 | 13.853 | 87.351 | 14.290 | 0.53 |
| Tyrosine | Tyr | Amino acids | 101.904 | 29.097 | 104.481 | 29.979 | 0.53 |
| Valine | Val | Amino acids | 194.624 | 69.205 | 199.740 | 69.903 | 0.76 |
| Leucine and isoleucine | xLeu | Amino acids | 230.361 | 68.048 | 233.301 | 65.945 | 0.40 |
| Lysophosphatidylcholine (acyl) C14:0 | lysoPC a C14:0 | Glycerophospholipids | 4.154 | 0.830 | 4.241 | 0.861 | 0.66 |
| Lysophosphatidylcholine (acyl) C16:0 | lysoPC a C16:0 | Glycerophospholipids | 87.575 | 25.186 | 92.592 | 26.148 | 0.66 |
| Lysophosphatidylcholine (acyl) C16:1 | lysoPC a C16:1 | Glycerophospholipids | 2.979 | 1.215 | 3.047 | 1.262 | 0.64 |
| Lysophosphatidylcholine (acyl) C17:0 | lysoPC a C17:0 | Glycerophospholipids | 1.479 | 0.559 | 1.576 | 0.578 | 0.67 |
| Lysophosphatidylcholine (acyl) C18:0 | lysoPC a C18:0 | Glycerophospholipids | 24.525 | 7.267 | 26.298 | 7.732 | 0.59 |
| Lysophosphatidylcholine (acyl) C18:1 | lysoPC a C18:1 | Glycerophospholipids | 19.749 | 6.928 | 19.722 | 6.827 | 0.59 |
| Lysophosphatidylcholine (acyl) C18:2 | lysoPC a C18:2 | Glycerophospholipids | 34.503 | 13.497 | 32.861 | 12.823 | 0.51 |
| LysoPhosphatidylcholine (acyl) C20:3 | lysoPC a C20:3 | Glycerophospholipids | 2.516 | 0.928 | 2.463 | 0.907 | 0.50 |
| Lysophosphatidylcholine (acyl) C20:4 | lysoPC a C20:4 | Glycerophospholipids | 6.221 | 2.239 | 6.374 | 2.272 | 0.60 |
| Phosphatidylcholine (diacyl) C26:0 | PC aa C26:0 | Glycerophospholipids | 0.765 | 0.234 | 0.751 | 0.212 | 0.77 |
| Phosphatidylcholine (diacyl) C28:1 | PC aa C28:1 | Glycerophospholipids | 3.669 | 1.067 | 3.715 | 1.088 | 0.68 |
| Phosphatidylcholine (diacyl) C30:0 | PC aa C30:0 | Glycerophospholipids | 6.303 | 2.249 | 6.212 | 2.201 | 0.52 |
| Phosphatidylcholine (diacyl) C32:0 | PC aa C32:0 | Glycerophospholipids | 15.343 | 4.045 | 15.289 | 3.994 | 0.62 |
| Phosphatidylcholine (diacyl) C32:1 | PC aa C32:1 | Glycerophospholipids | 21.035 | 12.424 | 20.105 | 11.561 | 0.65 |
| Phosphatidylcholine (diacyl) C32:2 | PC aa C32:2 | Glycerophospholipids | 5.545 | 2.046 | 5.342 | 2.020 | 0.58 |
| Phosphatidylcholine (diacyl) C32:3 | PC aa C32:3 | Glycerophospholipids | 0.629 | 0.218 | 0.590 | 0.217 | 0.73 |
| Phosphatidylcholine (diacyl) C34:1 | PC aa C34:1 | Glycerophospholipids | 248.360 | 75.809 | 246.062 | 75.288 | 0.67 |
| Phosphatidylcholine (diacyl) C34:2 | PC aa C34:2 | Glycerophospholipids | 412.423 | 117.111 | 403.814 | 117.644 | 0.76 |
| Phosphatidylcholine (diacyl) C34:3 | PC aa C34:3 | Glycerophospholipids | 20.555 | 6.765 | 19.460 | 6.636 | 0.56 |
| Phosphatidylcholine (diacyl) C34:4 | PC aa C34:4 | Glycerophospholipids | 2.620 | 1.017 | 2.535 | 1.028 | 0.63 |
| Phosphatidylcholine (diacyl) C36:0 | PC aa C36:0 | Glycerophospholipids | 3.305 | 1.040 | 3.259 | 1.031 | 0.62 |
| Phosphatidylcholine (diacyl) C36:1 | PC aa C36:1 | Glycerophospholipids | 61.339 | 18.044 | 60.143 | 18.443 | 0.55 |
| Phosphatidylcholine (diacyl) C36:2 | PC aa C36:2 | Glycerophospholipids | 266.484 | 71.358 | 260.866 | 72.706 | 0.69 |
| Phosphatidylcholine (diacyl) C36:3 | PC aa C36:3 | Glycerophospholipids | 164.250 | 44.026 | 159.723 | 43.770 | 0.65 |
| Phosphatidylcholine (diacyl) C36:4 | PC aa C36:4 | Glycerophospholipids | 221.742 | 63.711 | 220.563 | 63.707 | 0.74 |
| Phosphatidylcholine (diacyl) C36:5 | PC aa C36:5 | Glycerophospholipids | 39.387 | 20.338 | 38.809 | 19.936 | 0.48 |
| Phosphatidylcholine (diacyl) C36:6 | PC aa C36:6 | Glycerophospholipids | 1.529 | 0.674 | 1.466 | 0.644 | 0.57 |
| Phosphatidylcholine (diacyl) C38:0 | PC aa C38:0 | Glycerophospholipids | 3.300 | 1.086 | 3.239 | 1.091 | 0.66 |
| Phosphatidylcholine (diacyl) C38:3 | PC aa C38:3 | Glycerophospholipids | 63.003 | 17.728 | 62.403 | 17.857 | 0.63 |
| Phosphatidylcholine (diacyl) C38:4 | PC aa C38:4 | Glycerophospholipids | 125.897 | 36.329 | 126.014 | 36.572 | 0.71 |
| Phosphatidylcholine (diacyl) C38:5 | PC aa C38:5 | Glycerophospholipids | 65.426 | 19.544 | 64.717 | 19.488 | 0.62 |
| Phosphatidylcholine (diacyl) C38:6 | PC aa C38:6 | Glycerophospholipids | 107.315 | 34.644 | 105.734 | 34.582 | 0.63 |
| Phosphatidylcholine (diacyl) C40:1 | PC aa C40:1 | Glycerophospholipids | 0.402 | 0.103 | 0.398 | 0.102 | 0.47 |
| Phosphatidylcholine (diacyl) C40:2 | PC aa C40:2 | Glycerophospholipids | 0.381 | 0.126 | 0.377 | 0.130 | 0.32 |
| Phosphatidylcholine (diacyl) C40:3 | PC aa C40:3 | Glycerophospholipids | 0.681 | 0.211 | 0.675 | 0.218 | 0.39 |
| Phosphatidylcholine (diacyl) C40:4 | PC aa C40:4 | Glycerophospholipids | 4.153 | 1.397 | 4.107 | 1.371 | 0.62 |
| Phosphatidylcholine (diacyl) C40:5 | PC aa C40:5 | Glycerophospholipids | 14.106 | 4.631 | 14.064 | 4.607 | 0.62 |
| Phosphatidylcholine (diacyl) C40:6 | PC aa C40:6 | Glycerophospholipids | 38.411 | 13.609 | 38.085 | 13.792 | 0.63 |
| Phosphatidylcholine (diacyl) C42:0 | PC aa C42:0 | Glycerophospholipids | 0.594 | 0.206 | 0.591 | 0.207 | 0.7 |
| Phosphatidylcholine (diacyl) C42:1 | PC aa C42:1 | Glycerophospholipids | 0.290 | 0.091 | 0.289 | 0.089 | 0.67 |
| Phosphatidylcholine (diacyl) C42:2 | PC aa C42:2 | Glycerophospholipids | 0.279 | 0.086 | 0.272 | 0.082 | 0.47 |
| Phosphatidylcholine (diacyl) C42:4 | PC aa C42:4 | Glycerophospholipids | 0.188 | 0.049 | 0.188 | 0.050 | 0.53 |
| Phosphatidylcholine (diacyl) C42:5 | PC aa C42:5 | Glycerophospholipids | 0.483 | 0.170 | 0.480 | 0.176 | 0.43 |
| Phosphatidylcholine (diacyl) C42:6 | PC aa C42:6 | Glycerophospholipids | 0.618 | 0.191 | 0.606 | 0.188 | 0.54 |
| Phosphatidylcholine (acyl-alkyl)C30:0 | PC ae C30:0 | Glycerophospholipids | 0.419 | 0.149 | 0.416 | 0.147 | 0.59 |
| Phosphatidylcholine (acyl-alkyl) C30:2 | PC ae C30:2 | Glycerophospholipids | 0.187 | 0.068 | 0.188 | 0.071 | 0.78 |
| Phosphatidylcholine (acyl-alkyl) C32:1 | PC ae C32:1 | Glycerophospholipids | 2.843 | 0.749 | 2.835 | 0.761 | 0.65 |
| Phosphatidylcholine (acyl-alkyl) C32:2 | PC ae C32:2 | Glycerophospholipids | 0.725 | 0.215 | 0.720 | 0.224 | 0.73 |
| Phosphatidylcholine (acyl-alkyl) C34:0 | PC ae C34:0 | Glycerophospholipids | 1.797 | 0.591 | 1.788 | 0.607 | 0.61 |
| Phosphatidylcholine (acyl-alkyl) C34:1 | PC ae C34:1 | Glycerophospholipids | 10.707 | 2.977 | 10.684 | 3.074 | 0.64 |
| Phosphatidylcholine (acyl-alkyl) C34:2 | PC ae C34:2 | Glycerophospholipids | 12.437 | 3.832 | 12.158 | 3.836 | 0.61 |
| Phosphatidylcholine (acyl-alkyl) C34:3 | PC ae C34:3 | Glycerophospholipids | 8.151 | 2.649 | 8.043 | 2.690 | 0.69 |
| Phosphatidylcholine (acyl-alkyl) C36:0 | PC ae C36:0 | Glycerophospholipids | 1.010 | 0.306 | 0.999 | 0.333 | 0.63 |
| Phosphatidylcholine (acyl-alkyl) C36:1 | PC ae C36:1 | Glycerophospholipids | 8.708 | 2.529 | 8.709 | 2.641 | 0.65 |
| Phosphatidylcholine (acyl-alkyl) C36:2 | PC ae C36:2 | Glycerophospholipids | 16.421 | 4.829 | 16.177 | 4.931 | 0.69 |
| Phosphatidylcholine (acyl-alkyl) C36:3 | PC ae C36:3 | Glycerophospholipids | 9.329 | 2.733 | 9.053 | 2.716 | 0.62 |
| Phosphatidylcholine (acyl-alkyl) C36:4 | PC ae C36:4 | Glycerophospholipids | 19.472 | 6.213 | 19.120 | 5.744 | 0.64 |
| Phosphatidylcholine (acyl-alkyl) C36:5 | PC ae C36:5 | Glycerophospholipids | 13.740 | 4.172 | 13.705 | 4.088 | 0.68 |
| Phosphatidylcholine (acyl-alkyl) C38:0 | PC ae C38:0 | Glycerophospholipids | 2.386 | 0.868 | 2.282 | 0.838 | 0.61 |
| Phosphatidylcholine (acyl-alkyl) C38:2 | PC ae C38:2 | Glycerophospholipids | 2.389 | 0.630 | 2.354 | 0.654 | 0.57 |
| Phosphatidylcholine (acyl-alkyl) C38:3 | PC ae C38:3 | Glycerophospholipids | 4.714 | 1.276 | 4.656 | 1.287 | 0.64 |
| Phosphatidylcholine (acyl-alkyl) C38:4 | PC ae C38:4 | Glycerophospholipids | 13.957 | 3.649 | 13.916 | 3.628 | 0.66 |
| Phosphatidylcholine (acyl-alkyl) C38:5 | PC ae C38:5 | Glycerophospholipids | 20.108 | 5.279 | 19.872 | 5.172 | 0.65 |
| Phosphatidylcholine (acyl-alkyl) C38:6 | PC ae C38:6 | Glycerophospholipids | 8.660 | 2.552 | 8.477 | 2.509 | 0.6 |
| Phosphatidylcholine (acyl-alkyl) C40:0 | PC ae C40:0 | Glycerophospholipids | 6.254 | 1.864 | 6.000 | 1.789 | 0.67 |
| Phosphatidylcholine (acyl-alkyl) C40:1 | PC ae C40:1 | Glycerophospholipids | 1.449 | 0.419 | 1.411 | 0.413 | 0.6 |
| Phosphatidylcholine (acyl-alkyl) C40:2 | PC ae C40:2 | Glycerophospholipids | 2.204 | 0.630 | 2.187 | 0.644 | 0.66 |
| Phosphatidylcholine (acyl-alkyl) C40:3 | PC ae C40:3 | Glycerophospholipids | 1.108 | 0.267 | 1.095 | 0.274 | 0.65 |
| Phosphatidylcholine (acyl-alkyl) C40:4 | PC ae C40:4 | Glycerophospholipids | 2.569 | 0.627 | 2.554 | 0.629 | 0.64 |
| Phosphatidylcholine (acyl-alkyl) C40:5 | PC ae C40:5 | Glycerophospholipids | 4.114 | 0.999 | 4.013 | 0.976 | 0.62 |
| Phosphatidylcholine (acyl-alkyl) C40:6 | PC ae C40:6 | Glycerophospholipids | 5.555 | 1.669 | 5.475 | 1.645 | 0.66 |
| Phosphatidylcholine (acyl-alkyl) C42:0 | PC ae C42:0 | Glycerophospholipids | 0.490 | 0.104 | 0.486 | 0.103 | 0.56 |
| Phosphatidylcholine (acyl-alkyl) C42:1 | PC ae C42:1 | Glycerophospholipids | 0.438 | 0.120 | 0.433 | 0.118 | 0.61 |
| Phosphatidylcholine (acyl-alkyl) C42:2 | PC ae C42:2 | Glycerophospholipids | 0.684 | 0.188 | 0.675 | 0.188 | 0.53 |
| Phosphatidylcholine (acyl-alkyl) C42:3 | PC ae C42:3 | Glycerophospholipids | 0.792 | 0.211 | 0.779 | 0.214 | 0.59 |
| Phosphatidylcholine (acyl-alkyl) C42:4 | PC ae C42:4 | Glycerophospholipids | 0.974 | 0.270 | 0.970 | 0.269 | 0.64 |
| Phosphatidylcholine (acyl-alkyl) C42:5 | PC ae C42:5 | Glycerophospholipids | 2.262 | 0.578 | 2.222 | 0.566 | 0.69 |
| Phosphatidylcholine (acyl-alkyl) C44:3 | PC ae C44:3 | Glycerophospholipids | 0.135 | 0.039 | 0.134 | 0.038 | 0.57 |
| Phosphatidylcholine (acyl-alkyl) C44:4 | PC ae C44:4 | Glycerophospholipids | 0.422 | 0.125 | 0.422 | 0.124 | 0.66 |
| Phosphatidylcholine (acyl-alkyl) C44:5 | PC ae C44:5 | Glycerophospholipids | 1.932 | 0.611 | 1.949 | 0.612 | 0.72 |
| Phosphatidylcholine (acyl-alkyl) C44:6 | PC ae C44:6 | Glycerophospholipids | 1.274 | 0.405 | 1.285 | 0.405 | 0.71 |
| Hydroxysphingomyeline C14:1 | SM (OH) C14:1 | Sphingolipids | 8.167 | 2.991 | 8.366 | 3.077 | 0.80 |
| Hydroxysphingomyeline C16:1 | SM (OH) C16:1 | Sphingolipids | 4.451 | 1.607 | 4.546 | 1.670 | 0.79 |
| Hydroxysphingomyeline C22:1 | SM (OH) C22:1 | Sphingolipids | 17.784 | 6.306 | 17.878 | 6.432 | 0.78 |
| Hydroxysphingomyeline C22:2 | SM (OH) C22:2 | Sphingolipids | 14.986 | 5.528 | 15.221 | 5.704 | 0.81 |
| Sphingomyeline C16:0 | SM C16:0 | Sphingolipids | 129.334 | 35.575 | 130.910 | 36.028 | 0.75 |
| Sphingomyeline C16:1 | SM C16:1 | Sphingolipids | 21.111 | 6.257 | 21.295 | 6.277 | 0.77 |
| Sphingomyeline C18:0 | SM C18:0 | Sphingolipids | 33.795 | 10.004 | 34.171 | 10.180 | 0.74 |
| Sphingomyeline C18:1 | SM C18:1 | Sphingolipids | 15.847 | 5.293 | 15.840 | 5.306 | 0.79 |
| Sphingomyeline C24:0 | SM C24:0 | Sphingolipids | 29.439 | 9.585 | 28.959 | 9.400 | 0.76 |
| Sphingomyeline C24:1 | SM C24:1 | Sphingolipids | 72.863 | 21.818 | 72.622 | 22.000 | 0.73 |
| Hexose | H1 | Sugars | 6599.380 | 2017.040 | 6404.350 | 2060.110 | 0.55 |
